# Development of Sensor Registry System-Based Predictive Information Service Using a Grid

**DOI:** 10.3390/s18113620

**Published:** 2018-10-25

**Authors:** Hyunjun Jung, Dongwon Jeong, Sukhoon Lee

**Affiliations:** 1 Sensor Intelligence Research Center, Gwangju Institute of Science and Technology, Buk-gu, Gwangju 61005, Korea; junghj85@gist.ac.kr; 2 Department of Software Convergence Engineering, Kunsan National University, Gunsan-si, Jeollabuk-do 54150, Korea

**Keywords:** sensor registry system, path prediction, mobile network coverage, received signal strength, network coverage grouping

## Abstract

A sensor registry system (SRS) registers sensor metadata and provides them for a seamless semantic process. Recently, network coverage information-based SRS (NC-SRS) was developed to provide sensor information filtering by combining path prediction and network coverage checks. However, the NC-SRS has problems caused by issues such as termination of OpenSignal service and pre-building road segments. Therefore, this paper proposes a sensor registry system-based predictive information service (SRS-PIS) using a grid. SRS-PIS predicts a path based on the grid, checks the network coverage, and filters the sensor. This paper presents a grid-based real-time path prediction algorithm and an algorithm for grouping network service-disabled areas. To obtain network coverage information, we constructed and implemented a grid-based coverage map through experiment to measure the signal strength. As an evaluation, we compared features among SRS-based systems and SRS-PIS, and compared advantages and disadvantages between segment-based and grid-based methods.

## 1. Introduction

The evolution of Internet of Things (IoT) enables the data collected through an existing sensor network to be transmitted through the Internet [1,2]. In advancing cloud computing technology, cloud platforms that store sensor data in an IoT environment have been developed [3,4,5]. To store sensor data, an understanding of sensors and domain knowledge is required in various domains, and research studies have been conducted to model data on this knowledge and to build databases [6,7,8].

A sensor registry system (SRS) is based on the ISO/IEC 11179 metadata registry [9] for the management, sharing, and semantic processing of sensor metadata [10]. The SRS was originally developed for registering sensor metadata in a sensor network environment. However, in the IoT environment, the scope of application of the SRS has expanded in the mobile environment owing to the convenience of accessing sensor data.

An SRS sends information about the associated sensors to provide the user with the desired service. In this case, sensor filtering technology is required to selectively provide information from a large number of sensors registered in the SRS in a mobile environment with limited resources [11]. Therefore, a path prediction-based sensor registry system (PP-SRS) was proposed to predict user paths based on the GPS position of the user and to filter the sensor effectively [12]. The PP-SRS requires a dynamic path prediction algorithm and predicts in which direction the user will move. Sensor information around the predicted path is then provided in real time from the SRS.

However, in an area where the network connection is unstable and mobile data communication is impossible, there is a problem in that sensor information cannot be received from the SRS even if the user paths are predicted. To solve this problem, a network coverage information-based sensor registry system (NC-SRS) has been researched [13]. It uses a network coverage map [14] to identify unstable areas in advance, and proposes a method of providing sensor information before moving the areas.

The NC-SRS has several functions such as path identification, network coverage checking, and sensor information matching and uses the elements of PP-SRS to perform path prediction. The NC-SRS uses road segments for path prediction. A road segment is a unit of roads that are fragmented by crossroads or the ends of the roads. However, the segment-based method has a disadvantage in that the road and segment information is pre-defined. The segment-based method also expresses the road on a map as a line and recognizes a user trajectory as a continuous line projected on a segment. However, the line expression of an area such as a broad road or a square has a limitation: representative segment identification and pattern learning in the area is particularly difficult. This is because the trajectory in the square is extracted as an abnormal segment pattern, such as moving to an unconnected segment, which is treated as noise.

To resolve the problems, we improve the NC-SRS using a grid. The grid is a unit for identifying a position and is a plane area divided by a rectangle on a map. It has an advantage in that the grid can be utilized in the path prediction using only the GPS position without pre-defining road information. This paper proposes a SRS-based predictive information service (SRS-PIS), and it predicts user paths and checks network coverage information to predictively select the sensors that the user will use. The information of selected sensors is received from the SRS and provided to the user. If the predicted path is included in a service-disabled area when the network coverage information is checked, SRS-PIS provides the information of all sensors in the service-disabled areas. Therefore, this paper proposes a grid-based path prediction algorithm and grid-grouping algorithms for checking network coverage information. For implementation and evaluation, we measured the signal strength in the target area and implemented a grid-grouping algorithm. We also compared the SRS-PIS with other systems such as the NC-SRS and demonstrate the contribution of the SRS-PIS.

The rest of this paper is structured as follows: Section 2 introduces related works about grid-based path prediction research and mobile crowdsensing approaches. Section 3 describes SRS-PIS as our proposed system with the path prediction algorithm and grid grouping algorithms, and Section 4 presents an experimental implementation and evaluation. Section 5 concludes the paper.

## 2. Related Works

### 2.1. Grid-Based Path Prediction

There have been various research studies about path prediction technologies. This section presents path prediction research based on a grid.

Continuous Route Pattern Mining (CRPM) identifies the user’s location using the GPS position as a cell-temporal sequence (CTS) and regional-temporal sequence (RTS) to predict the personal route [15]. In a grid, CTS and RTS are used to learn movement trajectories for each user. When a grid is created, its location is defined using the combined trajectories of all users. However, the grid creation method has a problem in that it is impossible to process new data that are outside of the entire grid range.

R2-D2 is a semi-lazy approach that predicts a path using a small set of similar reference trajectories [16]. R2-D2 searches a similar trajectory using trajectories of vehicles in urban space, and each vehicle then identifies the location using a fixed grid based on its GPS position.

Subsyn proposed a destination prediction algorithm based on a Markov model [17]. It divides a map into grids using GPS positions, measures the probability of moving from one grid to the next, and predicts user paths using a Markov model and Bayesian inference. Subsyn claims that using a grid is effective for protecting user privacy because a location is abstracted into an area and the exact position is not exposed.

Wen He et al. developed a ridesharing recommendation system by learning the individual trajectories of a user [18]. The system identifies a movement trajectory using a grid and learns the regular routes of the user. It then groups the users by their regular routes and recommends the users to each other for ridesharing. This research showed how to create a grid from user trajectories and to find similar trajectories instead of describing a path prediction algorithm.

Hidden Markov model-based Trajectory Prediction (HTMP) proposed a hidden Markov model (HMM)-based trajectory prediction algorithm [19]. It maps the user location into a grid and learns HMM with various features.

Various research studies have tried to predict user paths using a grid, but most of them did not guarantee path prediction in real time. PP-SRS [12] is designed to support path prediction in real time and uses a segment-based method. The grid-based path prediction algorithm for SRS is also researched [20]. This method supports a grid-based and real-time path prediction. However, the method only presents a prediction process and a concept of learning, although it focuses on differences between the segment and grid.

This paper improves the NC-SRS [13] to apply a grid to a real-time path prediction algorithm. NC-SRS has the same path prediction algorithm as the PP-SRS. Thus, the grid-based path prediction algorithm described in [20] can be applied to the NC-SRS, but we also improve the algorithm by segmenting the weight by, for example, adding directionality to the grid.

### 2.2. Mobile Crowdsensing Approach

The proposed paper uses premeasured network coverage information to determine the communication status of each grid. Network coverage information combines mobile data measured by multiple users to determine the enabled status of a grid. The IoT environment has become common, and environmental data information is collected and new information is derived using sensors in mobile phones. This is called mobile crowdsensing [21]. Mobile crowdsensing is a concept that enables the use of mobile devices and ubiquitous characteristics to capture phenomena of common interest [22].

OpenSignal [14] measures the mobile network environment and gathers information collected by multiple users. It also provides integrated information through Web pages. Coverage maps for major networks around the globe are available for free in the OpenSignal app. OpenSignal does not reflect coverage in real time, but it can check the coverage situation in the approximate area.

Urban WiFi [23] characterization leverages the mobility of smartphones and people to present urban WiFi characteristics. Urban WiFi detects WiFi AP based on measurements taken by users in Edinburgh. Urban WiFi can determine the distribution of 2.4 GHz and 5 GHz channels through measurement, and spatial analysis of spectrum utilization is possible.

McSense [24] is a functional global warming platform for smart cities. McSense detects crowds throughout cities that are expected to be dense with its distributed architecture and data analytics. The main goal is to automate the organization of spontaneous and impromptu collaborations of large groups of people participating in collective actions.

IncentMe [25] proposes a framework that solves fundamental problems by leveraging game-theoretical reverse auction mechanism design. IncentMe is a road traffic monitoring application using mobile taxi tracks in San Francisco, Rome, and Beijing.

## 3. The Proposed Method

This section proposes the SRS-based predictive information system (SRS-PIS). The proposed method improves the existing NC-SRS and uses the grid. First, we describe how this research has evolved from the sensor registry system and what problems exist in the research, and describe the key concepts and processes of the proposed SRS-PIS. We then present the main algorithms that must be improved to apply the grid: a path prediction algorithm and a grouping algorithm for a service-disabled area.

### 3.1. Problem Statement

The SRS is a system for registering and sharing sensor information [9]. The SRS registers the meaning of sensor information such as sensor type, location, and unit of sensor value, so it can be used in a heterogeneous sensor network or an IoT environment. When the user requests the desired sensor information, the SRS finds and returns the registered sensor information, and this information is used to provide seamless services such as location-based services through semantic processing.

**Figure 1:**
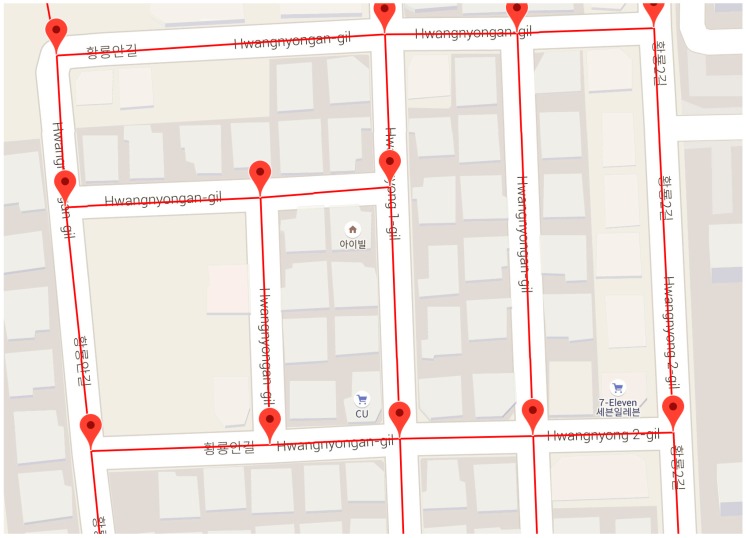
Expressed road segments on a map.

On the other hand, the development of smart phones required a way to effectively utilize the SRS in mobile environments. The SRS is structurally connected via the Internet, and the user requests and receives information about the sensor required for the service from the SRS. However, the PP-SRS has been proposed to provide sensor information in a more efficient manner [12]. The PP-SRS provides the sensor information located in the user location and the predicted path using a GPS position. At this time, the mobile device selects and uses only the sensor information necessary for the service among the sensor information previously provided from the SRS. This reduces the time required for the mobile device to know the current location of the user and select the required sensor and then request and receive sensor information from the SRS. The PP-SRS uses map road information called segments for current location identification, path learning, and prediction. Road segments are fragments of crossroad separation of each road, as shown in Figure 1.

**Figure 2:**
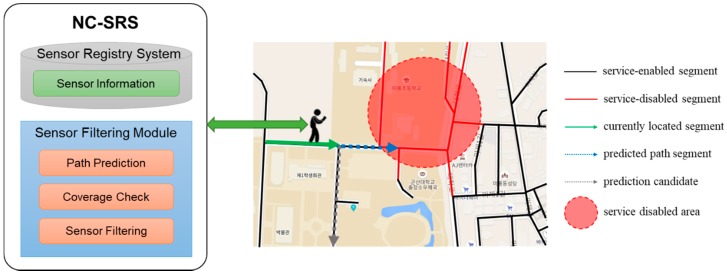
Concept of the network coverage information-based sensor registry system (NC-SRS) using a segment.

Since the PP-SRS is dependent on the mobile network, sensor information cannot be provided from the SRS when the mobile device cannot be stably connected to the network. In other words, when a user enters a network service-disabled area, the mobile device cannot receive sensor information about the current location as well as sensor information about the predicted path. This is critical to service provision based on sensor information, because the semantic processing of sensor information is impossible. Therefore, the NC-SRS was developed using the signal strength of the network [13]. The NC-SRS utilizes OpenSignal [14], which collects data by crowdsensing and serves Open API, to obtain network coverage information. The NC-SRS periodically obtains the network signal strength of each region from OpenSignal and determines whether the road segment belongs to a network service-disabled area. Figure 2 shows the main concept of the NC-SRS using a segment. If the NC-SRS predicts that the user will enter the service-disabled area, the NC-SRS provides all the sensor information belonging to the service-disabled area to the user in advance. In this way, the sensor information can be received from the SRS in advance even if the mobile device cannot access the network, so that the user can receive the seamless service.

Before the problems of this research are addressed, we need to mention the characteristics of our research. This research is based on a real-time system. The mobile device must predict the path in real-time according to the user’s current location. Even if the prediction result is wrong, it should be possible to modify the prediction result dynamically. In another characteristic, this research may not be important in the accuracy of the path prediction. In other words, even if the path prediction result is wrong, the system can predict the path again and receive the sensor information from the SRS, and this only requires more time for the mobile device to obtain the needed sensor information.

With these characteristics, the NC-SRS has several problems. First, the NC-SRS has to receive the signal strength periodically and determines the service-disabled area. However, due to the termination of the service of OpenSignal, which provides the signal strength, the checking network coverage becomes impossible. Therefore, an alternative is required to obtain signal strength by replacing OpenSignal. This paper attempts to solve the problem by collecting signal strengths together when collecting user locations for path learning.

Second, both the PP-SRS and the NC-SRS use road segments for identifying paths and checking disabled-service areas. However, the segment-based method has a problem that is necessary to pre-define the road information that can be moved by the vehicle and the user. In other words, the segment-based method requires a high cost to construct road information, such as the location of the road point, the line indicating the road, and the connection points of the respective roads. If the service provider wishes to extend the coverage area or move the service to another area, the road information should be redefined with respect to the corresponding area. In addition, the segment-based method cannot be covered movement across open areas such as a square. If a user crosses the square, the segment-based method is considered to be moving on unconnected roads, which causes noise generation. This paper tries to solve the problems by using a grid based on the GPS positions composed of latitude and longitude.

### 3.2. The Sensor-Registry-System-Based Predictive Information Service

To solve the problems, this paper improves the NC-SRS by using a grid instead of a road segment. SRS-PIS as a proposed system uses similar methods and processes from the NC-SRS. The SRS-PIS also uses functions such as path prediction, network coverage checking, and sensor filtering to obtain the sensor information from SRS in advance. Through the SRS-PIS, a user can provide information of all sensors mapped in a currently located grid, a predicted path grid, and a group of service-disabled grids where the predicted path grid is the service-disabled grid.

**Figure 3:**
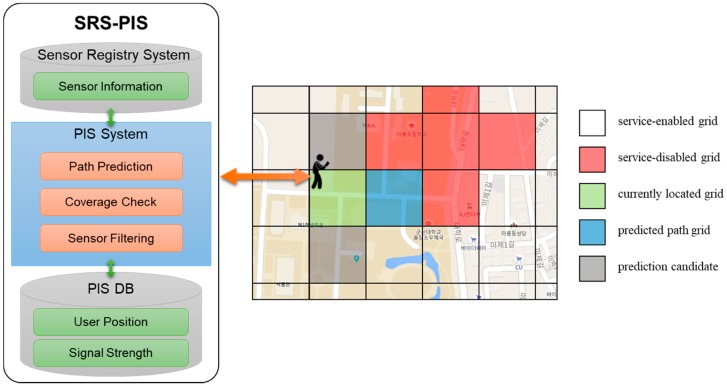
Concept of the SRS-based predictive information service (SRS-PIS) using a grid.

Figure 3 shows the main concept of the SRS-PIS using a grid. The PIS has several functions such as path prediction, network coverage check, and sensor filtering. The user obtains sensor information by linking SRS as a sensor filtering result. The SRS-PIS also has a database (PIS DB) that collects user position and signal strength of the position. The SRS just registers and manages sensor information, and it is not related to path prediction and coverage check. Thus, the SRS and the PIS database are divided for implementation.

A grid is a unit dividing an area into map locations and is defined as a fixed-size rectangular plane. There is no preconstruction cost because the grid is constructed with only the coordinates of the target area and the size of the grid. The SRS-PIS has an advantage in that it can flexibly process when a user moves off a road because it regards the user path as the connection of the plains rather than the lines.

The process of the SRS-PIS is as follows:

(1) Fetch user location: The user location is read from a smartphone or a wearable device and stored to a server.
(2) Predict user paths: Path prediction is performed using collected user locations. As a prediction result, a grid that has a high probability is selected from grids that are adjacent to the currently located grid for a user.
(3) Check enabled network coverage: That the predicted grid enables a network service is checked. If the predicted grid is in a service-disabled area, the predicted grid is expanded to include a service-disabled grid group.
(4) Filter sensors: The list of sensors that match the predicted grid registered in the SRS is extracted.
(5) Provide matched sensor information: The user is provided with the information of sensors in the list from the SRS.

As a result, the SRS-PIS provides information about the sensors located around the user and the sensors around the user’s movement path in a real-time environment. In this process, two tasks such as grid-based path prediction and network coverage checks are required. First, the user performs the task of predicting the user path by sending the current location to the PIS. The PIS derives the currently located grid for the user and returns the next grid to which the user will move as a prediction result. The PIS then checks whether the next grid is a grid identified as a service-disabled grid.

This is because, when the user enters the service-disabled area, the current path and the prediction result cannot be obtained by communicating with the SRS-PIS. The user can use only the sensor information previously provided from the SRS-PIS as a prediction result when the moved grid is the same as the path predicted from the previous grid and is in a service-disabled area. However, since the user cannot communicate with the SRS-PIS, the user cannot receive the path prediction and sensor information. Therefore, if a user moves within a service-disabled area, the user must receive sensor information from all service-disabled grids in the area at once. To do this, tasks are required such as a method for checking a service-disabled grid and a method for grouping adjacent service-disabled grids.

The grid-based path prediction methods are described in Section 3.3, and how to check if one grid belongs to a service-enabled grid and how to group service-disabled grids are presented in Section 3.4. The method of filtering the sensor is not specifically described since it is only necessary to check whether the position of the sensor registered in the SRS exists in the target grid.

### 3.3. The Path Prediction Algorithm

We described the grid-based path prediction algorithm in Section 2.1. However, because SRS-PIS requires a real-time path prediction algorithm, we use an algorithm that applies the collective behavior pattern-based path prediction (CBP-PP) algorithm [12], which is a conventional segment-based path prediction algorithm, to the grid.

The CBP-PP algorithm is a dynamic path prediction algorithm that can return lightweight and fast prediction results based on the greedy algorithm. The algorithm is designed to predict the path in real time using fewer resources in the mobile environment than the accuracy of the prediction result. In addition, the CBP-PP algorithm is based on the concept of CBP in the path prediction. The path prediction algorithm proposed in this paper is also based on CBP, and CBP is effective for the path that the user first goes to, which means the path where there is not enough data to learn.

Meanwhile, Jung et al. [20] proposed a grid-based path prediction method by improving PP-SRS. This method is based on the Greedy algorithm, similar to the CBP-PP. However, the method is different from the CBP-PP algorithm in such factors as the absence of CBP concept, the usage of undirected weight, the expression of equations. Therefore, this paper focuses on the CBP-PP algorithm, which can express weight in more detailed equations and use directed weight.

The CBP-PP algorithm learns segment patterns of paths from all users based on segments and performs the prediction using the segment patterns. The algorithm identifies a currently located segment from the user location using predefined segments (road information) and learns which identified segments have been moved according to the time sequence. The user path is then predicted using the current position of the user and the learned pattern. The proposed path prediction algorithm is almost the same as the CBP-PP algorithm, and the segment is changed to a grid.

**Figure 4:**
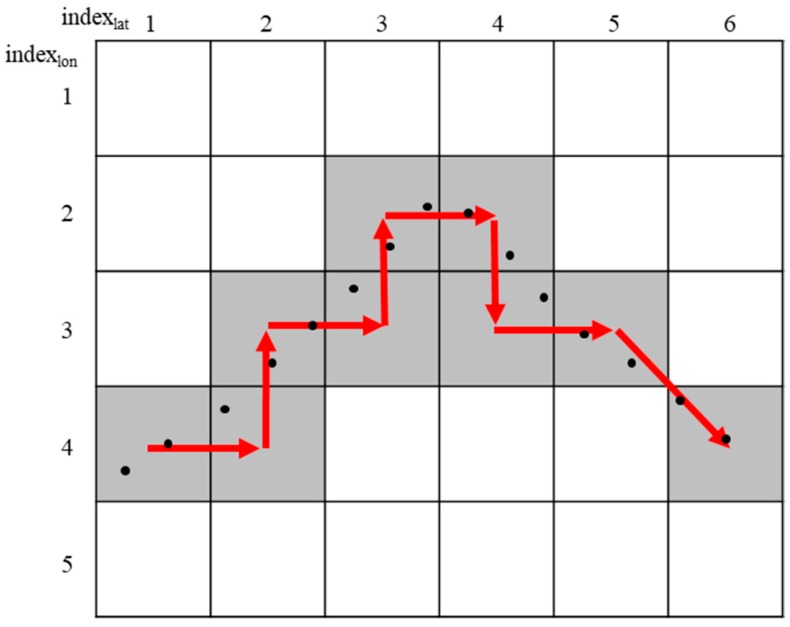
Grid-based path identification by user points.

Figure 4 shows how the grid is identified. The path pattern is created using the GPS coordinates of the users. Each point represents a user location, which consists of data of the (latitude, longitude) pair through a GPS position measurement. The user coordinate pair is mapped to grid G to obtain G_x,y_ = (x, y). At this time, the position and size of the grid are predefined. After that, the algorithm extracts paths such as Path = {G_x1,y1_, G_x2,y2_, …, G_xn,yn_} except for overlapping grids from each user location. In the case of Figure 4, the same grid sequence as Path_1_ = {(1, 4), (2, 4), (2, 3), (3, 3), (3, 2), (4, 2), (4, 3), (5, 3), (6, 4)} can be expressed as a path.

**Figure 5:**
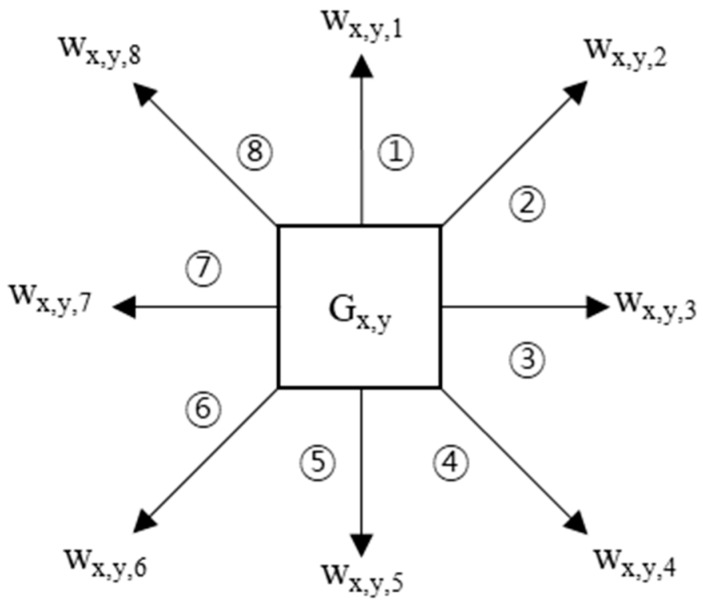
Weights of grid in eight directions.

For the learning paths, the algorithm measures weights as it passes from one grid to the next in each path. The CBP-PP has segments connected to each other based on the crossroad, but each segment has an independent weight. Figure 5 shows the weights of a grid in eight directions. The weights in path learning add the gain according to the direction of each grid when going from one grid to the next grid. For example, in Figure 4, the first two sequences {(1, 4), (2, 4)} move from G_1,4_ to G_2,4_, so they can be seen to move in direction ③. Therefore, this movement affects the weight w_x,y,3_.

Equation (1) shows how to calculate the weight in all directions in the grid G_x,y_:

(1) $$p(d\;|\;G_{x,y})=\frac{w_{x,y,d}}{\sum_{i=1}^{8}w_{x,y,i}}.$$

The algorithm then learns and calculates the weight according to the movement path for all Path_i_. The path prediction function is defined as Equation (2):

(2) $$\mathrm{prediction}(G_{x,y})=\mathrm{move}(G_{x,y},\;\underset{d}{\arg\;\max}\;p(d\;|\;G_{x,y}\cap d\neq \mathrm{source}(G_{x,y}))).$$

Prediction (G_x,y_) predicts which grid to move to when the user is currently in G_x,y_. The function move (G_x1,y1_, d) returns the destination grid G_x2,y2_ when moving in direction d from G_x1,y1_. The function source (G_xi,yi_) returns the direction d when move (G_xi−1,yi−1_, d) = G_xi,yi_. In other words, the function source returns what grid was moved before the currently identified grid.

The proposed path prediction algorithm predicts the short path of the next grid in the current grid as described above. The reason is that the SRS-PIS only uses the path prediction in real time as a method for filtering the sensor. Assuming that the real-time path is predicted and returned to a user, many mobile resources are required to determine the starting and ending points of the movement.

### 3.4. Grouping Service-Disabled Grids

**Figure 6:**
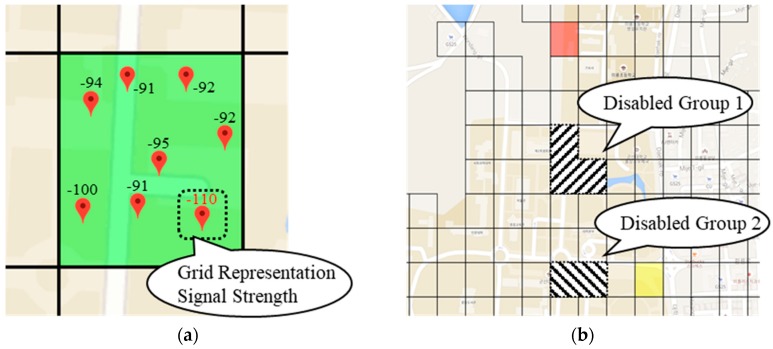
Examples of (**a**) determining service-disabled grid and (**b**) grouping service-disabled grid.

The purpose of grouping grids is to provide sensor information for a grid group with unstable signals by predicting the user’s path segment. The main idea of this paper is to collect signal strength information from users and use it to predict the signal conditions for each grid. The system sends the necessary sensor information in advance from the next grid if it predicts that the grid to be moved by the user will not be suitable to provide the service. Figure 6 shows the determination of a service-disabled grid and grouping service-disabled grid in the grid grouping process.

The grid grouping process is as follows:

(1) Set grid information: To determine the grid, the starting point (latitude and longitude), end point (latitude and longitude), and number of horizontal and longitudinal axes are set.
(2) Load strength information: The premeasured signal strength corresponding to the divided grid area is read.
(3) Determine service-disabled grid: The signal strength is stored as the representative signal strength, which is the weakest among the critical signal strengths in the grid, and a service-disabled grid is set. The signal strength threshold determines the service-disabled grid.
(4) Group service-disabled grids: To make adjacent grids, a group that has only service-disabled grids.
(5) Store the group number: The determined group number is stored in the grid information database.

The grid grouping process includes a disabled coverage measurement algorithm to determine the grid deliberated grid and service-disabled grid to combine them into a continuous grid.

Algorithm 1 shows the disabled configuration measurement algorithm. It accepts a set of signal strengths and a grid group from the measured signal strength that are below the threshold value of the lower signal strength. The algorithm checks whether the input signal strengths are included in all grid tours. If they are, the disabled status information is saved in the corresponding grid. The algorithm also saves the weakest signal strength as representative grid information and outputs the checked grid group. Algorithm 1 checks the number of grids (m) for the number of signal strength (n) at all measured points. However, since the number of grids does not increase, the time complexity of Algorithm 1 is O(n).

**Algorithm 1:** disabled_coverage_measuring (DS[], G[])
**Input:** set DS[]: disabled signal strength group, a grid group G[]
**Output:** set G[]: checked grid group1:**for each** G **do**2:      **for each** DS[]: ds **do**3:            **if** ds.GPS in G.range **then**4:                  g.Eabled = false5:                  g.Rep_strength = ds.strength6:            **endif**7:      **endfor**8:
**endfor**
9:**return** G[]

Algorithm 2 shows the disabled coverage grouping algorithm. It takes input from the output value of Algorithm 1, a group of checked grids. The algorithm identifies (top, bottom, left, and right) continuous grids among the entered disabled grids (DG[]) and number groups. It then prints the numbered disabled grid groups (DGG[][]). It then updates the disabled group number in the grid information database using the output. Algorithm 2 is based on a breadth first search (BFS) algorithm that groups the grids using queues. In general, the time complexity of the BFS algorithm is O (b^d+1^), where the branching factor is b and the search distance is d. In this paper, the branch of the grid is 4. The search distance is affected by the number of grids (n) from one end of the grid area to the other, so that the time complexity is O(4^n^).

**Algorithm 2:** disabled_coverage_grouping (DG[])
**Input:** set DG[] = {g1, g2, …, gm} of disabled grids
**Output:** set DGG[][] of disabled grid groups1:Let Q be a queue2:n ← 0    //group number3:**while** DG is not empty4:      g = DG.dequeue(0)5:      DGG[n].add(g)6:      Q.enqueue(g)7:      **while** Q is not empty8:            v = Q.dequeue()9:            Temp[] = DG[]10:            **for each** grid:Temp **do**11:                  **if**   isAdjacentGrid(v) **then** grid12:                        DGG[n].add(grid)13:                        Q.enqueue(grid)14:                        DG.remove(grid)15:                  **endif**16:            **endfor**17:      **endwhile**18:      n ← n + 119:
**endwhile**
20:**return** DGG[][]

## 4. Experimental Implementation and Evaluation

The SRS-PIS performs Algorithms 1 and 2 based on the network coverage information. These algorithms need signal strengths of mobile devices in each grid. In case of NC-SRS, it used open API of OpenSignal to obtain signal strengths, but the OpenSignal service is terminated for developers unfortunately. Therefore, we should directly measure network coverage information (location information, signal strength) for grouping service-disabled area.

OpenSignal determines signal strength as good or bad semantically by the values. However, Section 4.1 actually experiments with the data transmission rate according to the signal strength to determine the service-disabled area. Section 4.2 presents the grouping results using the signal strength collected by the Android app. Section 4.3 compares features among the SRS-based methods and SRS-PIS. We also compare the grid-based method with the segment-based method, and presented advantages of the grid-based method in Section 4.4.

### 4.1. Experiment of Data Transfer Rate

To measure the signal strength, we measure the received signal strength indicator (RSSI) of 3G and reference signals received power (RSRP) of 4G (LTE). The higher the signal strength, the faster the transmission speed. However, in order to know how much the data is actually affected by the communication, we experiment with the data transfer rate according to the signal strength.

To understand the standards of the communication environment in a 4G (LTE) environment, we measured the status of download, upload, and ping by varying the signal strength using BENCHBEE [26], which is an Android app. Table 1 lists the communication status by signal strength. At −120 dB (RSRP, 4G), it was not possible to measure downloads and uploads. At −115 dB to −110 dB (RSRP, 4G) download speeds were possible but were not uploaded. At −105 dB or more, the communication environment was inconvenient for us. At the end of the table, communication conditions were shown at a level of −110 dB for 4G from −81 dB, which is an environment where 3G works well. We anticipate that services will be difficult to use if the communications environment is between −120 dB and −105 dB.

### 4.2. Implementation and Evaluation of the Grouping Algorithm

**Figure 7:**
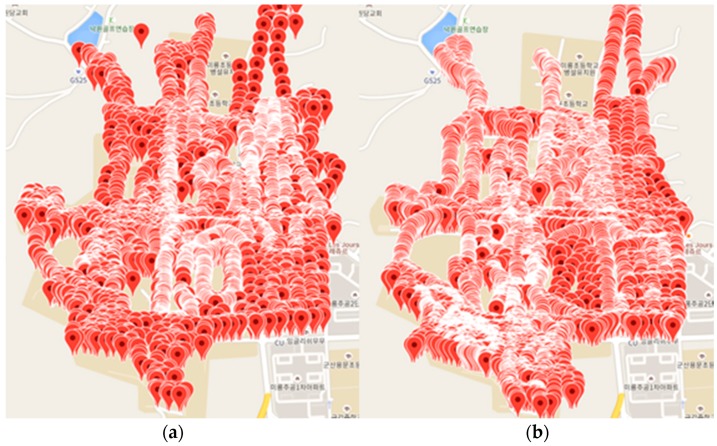
Results of measuring signal strength: (**a**) SKTelecom and (**b**) KT.

For implementation of Algorithms 1 and 2, we evaluate the actual collected signal strength and grouping results according to several environments. First, we collect GPS position and signal strength (4G, RSRP) using our developed Android app at a university and near the university area. At the time, the collected mobile network vendors are SKTelecom and KT in Korea. Figure 7a shows the measured signal strength spots of SKTelecom, and Figure 7b shows the spots of KT. We collected 110,762 SKTelecom spots and 112,031 KT spots for a total of 222,793 spots.

**Figure 8:**
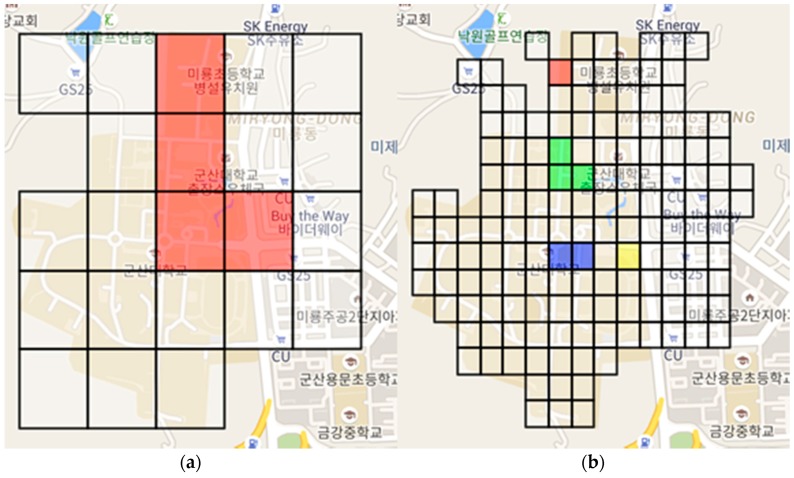
Teaming situation for SKTelecom: (**a**) 5 × 5 grids and (**b**) 15 × 15 grids.

**Figure 9:**
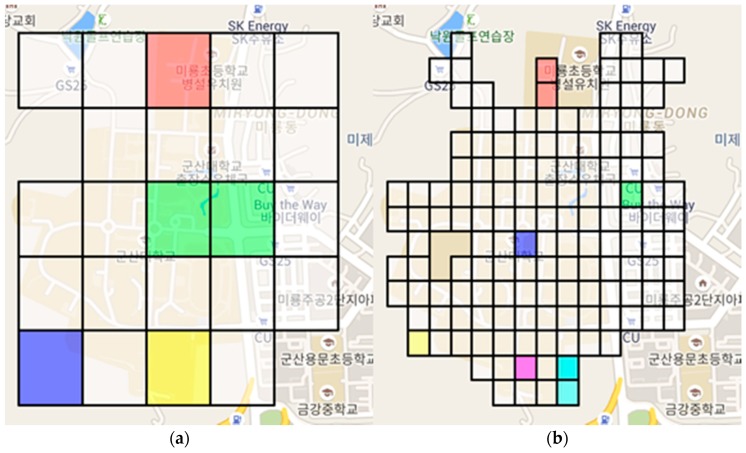
Teaming situation for KT: (**a**) 5 ×5 grids and (**b**) 15 × 15 grids.

Figure 8 shows a teaming situation for SKTelecom with the 5 × 5 grids and 15 × 15 grids. Figure 9 shows a teaming situation for KT with the 5 × 5 grids and 15 × 15 grids. The grid with a value below the threshold value among the signal strengths belonging to each grid is shown in color. The characteristics are different according to the size of grid applied to SRS-PIS. In other words, the colored grids are areas identified as a service-disabled area, and the same color is a group of service-disabled grids. If the size of the grid is large, the range of receiving the data is wide and the number of communication of the network is small. However, the user may receive additional sensor information that is not used. The smaller size of the grid can be a delicate disabled grouping. Therefore, the amount of sensor information received from the SRS is relatively small.

**Figure 10:**
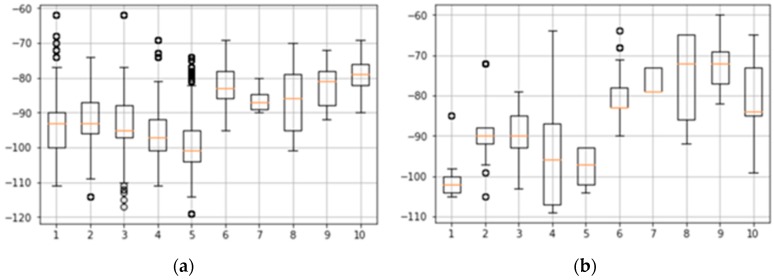
Box plots of signal strength per grid: (**a**) SKTelecom and (**b**) KT.

When a grid section is determined, the communication status of each grid is checked to determine if the grid is disabled. The disabled status of a grid is determined based on the signal strength. Communication failures occur when the signal strength value is between −120 dB and −105 dB in 4G (LTE). We decided that SKTelecom was a service-disabled grid when the data measured within a grid was −110 dB and KT −102 dB or less. Figure 10a shows a box plot of the signal strength per grid for SKTelecom, and Figure 10b shows a box plot for KT.

In each figure, numbers 1 to 5 of the *x*-axis indicate service-disabled grids, and numbers 6 to 10 of the *x*-axis indicate service-enabled grids. In the proposed method, the user selects the worst signal strength considering movement within the grid. However, it can be divided into service-disabled groups because of the rare signal strength. To address this, if the data patterns that occur in the service-enabled group and service-disabled group are determined based on the data patterns, it may be possible to maintain the service-disabled group more efficiently.

### 4.3. Comparison among SRS-Based Systems

This section compares systems based on the SRS and on this paper to verify their characteristics. The PP-SRS [12], the NC-SRS [13], Jung et al.’s system [20], and the SRS-PIS are selected for comparison. Table 2 shows the comparison result of the SRS-based systems and the SRS-PIS.

All systems support path prediction and can predictively provide sensor information. The PP-SRS and Jung et al.’s system support path prediction but do not support the network coverage method. This means that they are not guaranteed a reliable network connection that can be obtained through network coverage information. Moreover, Jung et al.’s system employs undirected weights, while other systems employ directed weights in each path prediction algorithm. The PP-SRS and the NC-SRS identify the user location with a segment-based structure. Jung et al.’s system and the SRS-PIS identify user location with a grid-based structure. The segment can be used only by pre-building road information, but the grid does not require pre-building because it can only be constructed under specified conditions, such as area range and the number of grids. In addition, the method of building segment information causes a high cost when the service is extended to another area. Finally, the PP-SRS and the NC-SRS load all or part of the road information into memory at system start-up, but Jung et al.’s system and the SRS-PIS do not need to load the grids. This causes a fast preprocessing performance.

### 4.4. Comparison between a Segment-Based Method and a Grid-Based Method

This section compares how the SRS-PIS, which is the grid-based method, is different from the NC-SRS, which is based on the segment. This paper compares the functional differences of the two systems in the process and explains the disadvantages of the segment-based path learning method as an example. Table 3 lists the results of a functional comparison. The characteristics for the comparison are defined as the path learning and prediction process, and the processes of the NC-SRS and the SRS-PIS are compared.

The tasks with the NC-SRS and the SRS-PIS show larger differences in preprocessing and path identification than in path learning, path prediction, or the results of path prediction. In particular, the NC-SRS investigates road information to build the segments. In this respect, quantitative evaluations such as building cost have already been presented in Ref. [20].

**Figure 11:**
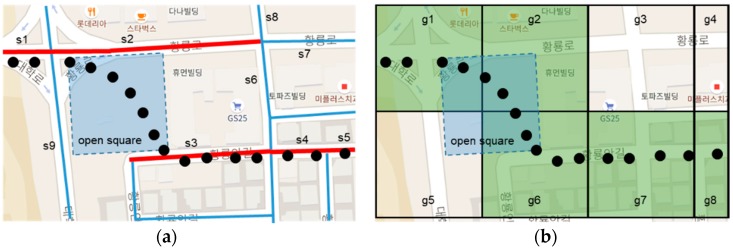
Result of path identification for trajectory passing through open square in (**a**) the NC-SRS and (**b**) the SRS-PIS.

As mentioned in Section 1, the segment-based method has the disadvantage that it expresses the movement of a plane such as a wide road or an open square and is difficult to learn because the map consists of lines. Figure 11 shows the path identification results for a trajectory passing through an open square in the segment-based method and the grid-based method. Since the open square can move in any direction when a person moves while walking, the segment-based method with lines finds it difficult to express movement on this plane. Figure 11a shows the path pattern of non-connected segments such as s1→s2→s3→s4→s5. When the user moves from s1 to s2, the system returns a prediction result as one of s6, s7, or s8. Thus, s3 cannot be returned as the prediction result in this case.

Figure 11b shows the identified path by the grid-based method. The trajectory is identified by a path pattern such as g1→g2→g6→g7→g8. In this case, a grid-based path pattern is more flexible because the learning and predicting paths are not related to the open square or road connection structure.

## 5. Conclusions

In order to provide sensor information efficiently, the NC-SRS, which uses path prediction and network coverage information, has been developed. However, OpenSignal service which provides signal strengths has been terminated, and the signal strengths must be required for checking the network coverage. Also the NC-SRS is a segment-based system that requires a high cost to build road segments. To resolve the problems, we proposed an SRS-based predictive information service, which is a grid-based system. To apply a grid to the proposed system, we also proposed a path prediction algorithm and a grouping disabled-service algorithm using the grid. We measured the signal strengths directly and used them for the network coverage checks to replace the OpenSignal. We also showed the contribution of SRS-PIS through a comparison with the PP-SRS, NC-SRS, and Jung et al.’s system.

SRS-PIS based on the grid replaces OpenSignal with a method of direct collecting the signal strength, so that data can be changed in real time and network coverage information can be reflected. This method is advantageous in that the signal strength can be collected together with the user’s GPS position, so any devices or apps are required separately. On the other hand, the grid-based method differs from the segment-based method in two ways. First, the segment-based method must require pre-building road information expressed by points and lines, but the grid-based method has no cost for the pre-building. Second, a trajectory in an area such as an open square is hard to be expressed as a segment, but the grid enables its expression.

The proposed system will be able to directly utilize more various and dense sensor information for service by the evolution of wearable device and IoT. It is also expected that, by applying a real-time system, the sensor information can be used for such services as street crime risk detection, driving alarms, and emergency rescues.

The grid used for the path prediction and the network coverage check does not need to be the same size. Therefore, research for designing and evaluating the range of the grid is required in the future. Additionally, since the sensor itself has a connection range, it is necessary to provide a service even to a sensor existing outside the target grid. Therefore, a sensor filtering method considering this will be developed.

## Figures and Tables

**Table 1:** Data transfer rate per signal strength.

Signal Strength	Download (Mbps)	Upload (Mbps)	PING (ms)
−120 dB (RSRP, 4G)	disabled	disabled	disabled
−115 dB (RSRP, 4G)	0.91	disabled	289
−110 dB (RSRP, 4G)	7.50	disabled	53.3
−105 dB (RSRP, 4G)	37.4	2.68	34.8
−100 dB (RSRP, 4G)	53.8	4.42	33.3
−86 dB (RSRP, 4G)	77.1	4.97	31.4
−81 dB (RSSI, 3G)	3.14	0.12	64.2

**Table 2:** PP-SRS, NC-SRS, Jung et al.’s system and SRS-PIS.

Feature	PP-SRS	NC-SRS	Jung et al.	SRS-PIS
path prediction	support	support	support	support
path prediction algorithm	directed weight	directed weight	undirected weight	directed weight
network coverage information	not support	support	not support	support
network stability	unstable	stable	unstable	stable
map structure type	segment	segment	grid	grid
map structure pre-building	need	need	not need	not need
area extension cost	high	high	low	low
preprocessing performance	medium	medium	fast	fast

**Table 3:** The PIS of the NC-SRS and the SRS-PIS processes.

Process	NC-SRS	SRS-PIS
preprocessing	loading all of roads that are manually defined.	creating grids automatically by geographical positions of target area.
path identification	identifying a currently located segment for a user by projecting a user position into the road expressed as a line.	identifying a currently located grid consisting of GPS position for a user.
path learning	learning the identified paths based on segments.	learning the identified paths based on grids.
path prediction algorithm	measuring frequency for segment weights with directions.	measuring frequency for grid weights with directions.
result of path prediction	a maximum weighted segment that is connected to a currently located segment.	a maximum weighted grid that is contiguous to a currently located grid.
